# Transcriptome Analysis of *Colletotrichum fructicola* Infecting *Camellia oleifera* Indicates That Two Distinct Geographical Fungi Groups Have Different Destructive Proliferation Capacities Related to Purine Metabolism

**DOI:** 10.3390/plants10122672

**Published:** 2021-12-05

**Authors:** Shimeng Tan, Yanying Chen, Guoying Zhou, Junang Liu

**Affiliations:** 1 Key Laboratory of National Forestry and Grassland Administration on Control of Artificial Forest Diseases and Pests in South China, Central South University of Forestry and Technology, Changsha 410004, China; sm_t0820@hotmail.com (S.T.); cyyanny@126.com (Y.C.); zgyingqq@163.com (G.Z.); 2 Hunan Provincial Key Laboratory for Control of Forest Diseases and Pests, Central South University of Forestry and Technology, Changsha 410004, China; 3 Key Laboratory for Non-Wood Forest Cultivation and Conservation of Ministry of Education, Central South University of Forestry and Technology, Changsha 410004, China; 4 College of Biological Science and Technology, Central South University of Forestry and Technology, Changsha 410004, China; 5 College of Forestry, Central South University of Forestry and Technology, Changsha 410004, China

**Keywords:** anthracnose of *Camellia oleifera*, *Colletotrichum fructicola*, transcriptome, differential expression, purine metabolism

## Abstract

Anthracnose, caused by *Colletotrichum* spp., is a significant disease affecting oil tea (*Camellia oleifera* Abel.). Extensive molecular studies have demonstrated that *Colletotrichum fructicola* is the dominant pathogen of oil tea anthracnose in China. This study aims to investigate differences in molecular processes and regulatory genes at a late stage of infection of *C. fructicola*, to aid in understanding differences in pathogenic mechanisms of *C. fructicola* of different geographic populations. We compared the pathogenicity of *C. fructicola* from different populations (Wuzhishan, Hainan province, and Shaoyang, Hunan province) and gene expression of representative strains of the two populations before and after inoculation in oil tea using RNA sequencing. The results revealed that *C. fructicola* from Wuzhishan has a more vital ability to impact oil tea leaf tissue. Following infection with oil tea leaves, up-regulated genes in the strains from two geographic populations were associated with galactosidase activity, glutamine family amino acid metabolism, arginine, and proline metabolism. Additionally, up-regulated gene lists associated with infection by Wuzhishan strains were significantly enriched in purine metabolism pathways, while Shaoyang strains were not. These results indicate that more transcriptional and translational activity and the greater regulation of the purine metabolism pathway in the *C. fructicola* of the Wuzhishan strain might contribute to its stronger pathogenicity.

## 1. Introduction

Oil tea (*Camellia oleifera*) is an agronomically and culturally important edible woody oil tree species found in China. Oil extracted from the seeds of oil tea is comparable to olive oil (*Olea europaea* L.). Oil tea is widely distributed between the northern latitudes of 32°57′ and 11°17′ in China [1]. Suitable climates for the growth of oil tea are generally mild, receive sufficient sunshine and rain, and have short or no ice periods. Therefore, the occurrence of disease and damage due to insect pests are frequent [2]. Anthracnose is a major disease of oil tea. Symptoms of the disease include leaf fall, flower fall, and fruit damage, which can impact yield or oil quality, leading to economic loss. It is caused by several species of *Colletotrichum* [3].

*Colletotrichum* species are globally distributed plant pathogens of many herbaceous and tree crops [4,5,6,7,8,9,10,11,12,13]. They are hemibiotrophic plant fungi and can affect almost all crops and economic plants. *Colletotrichum* spp. is often spread as conidia through wind, rain, insects, which means temperature and humidity will affect their spread and invasion [14]. The infection of plants by hemibiotrophic vegetative fungi is a progressive process whereby the infection of plants by *Colletotrichum* fungi can be divided into two stages: vegetative and death phase. After invading the host, mycelium of *Colletotrichum* grows intercellularly and kills the plant tissues (necrotic phase) [15,16]. In the later stage of the infection, the growth of the hypha and the acceleration of reproduction are depended on nutritional availability [17]. The former study has shown that climatic factors for the transmission and infestation directly affect the pattern of reproduction and spread in plant tissues [18]. Transcriptome sequencing can be used to study whether there are differences in the molecular regulation of *C. fructicola* from different geographical sources infecting oil tea, thereby deepening the understanding of the spreading mechanism of *C. fructicola* under different climatic conditions.

Eight species of anthracnose pathogens of oil tea have been identified based on morphological and polygenic molecular identification [19,20,21]. *Colletotrichum fructicola* is one of the prevalent *Colletotrichum* species associated to oil tea anthracnose in China and the most frequently isolated from leaves of oil tea [22]. Therefore, in this study we analyzed *C. fructicola* strains isolated sourced in Shaoyang, Hunan province, and Wuzhishan, Hainan province, China, respectively, using transcriptomic analysis. Focused on the transcript comparison of later infection stages of *C. fructicola* from different geographic sources, we hope to improve our understanding of the molecular mechanisms of pathogenesis of *C. fructicola* under different climate conditions. Transcriptome analysis of infection of *Camellia oleifera* by *C. fructicola* indicates that two distinct geographical groups have different destructive proliferation capacities related to purine metabolism.

## 2. Results

### 2.1. Comparison of Pathogenicity of Two Distinct Populations

**Figure 1:**
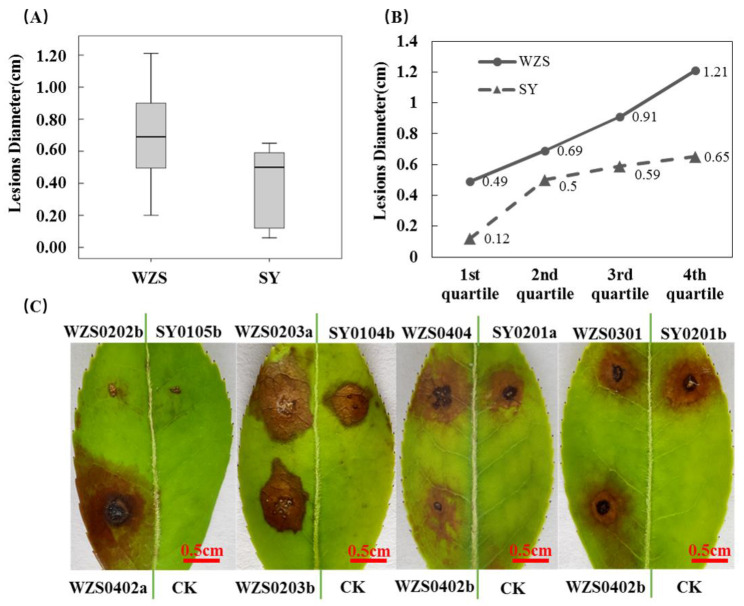
Pathogenicity of *C. fructicola* populations collected from Shaoyang and Wuzhishan to oil-tea leaves. CK is for control that infects leaves with pure water. (**A**) Lesion diameters 96 hpi (hours post infection) on oil-tea leaves following inoculation with *C. fructicola* populations from Wuzhishan and Shaoyang. (**B**) Quartiles of Lesion diameters 96 hpi on oil-tea leaves following inoculation with *C. fructicola* populations from Wuzhishan and Shaoyang, respectively. (**C**) General view of Lesions 96 hpi (one of three biological replicates). Green vertical lines represent leaf veins. Wuzhishan strains and Shaoyang strains were inoculated on the left and right sides of the leaves.

Diameter of lesions after *C. fructicola* inoculation was measured, and the results are presented in Figure 1. The mean diameter of lesions following incubation for the Shaoyang population, was 0.50 cm, while for the Wuzhishan population it was 0.69 cm. According to the Mann–Whitney *U* test, differences between the two populations were significant (*p* = 4.248 × 10^−7^ < 0.05). 

### 2.2. Comparison of Pathogenicity within Populations and Selection of Representative Strains

A comparative test of pathogenicity among the strains in the two populations was performed to select a representative strain from each of the two populations for transcriptome analysis.

**Figure 2:**
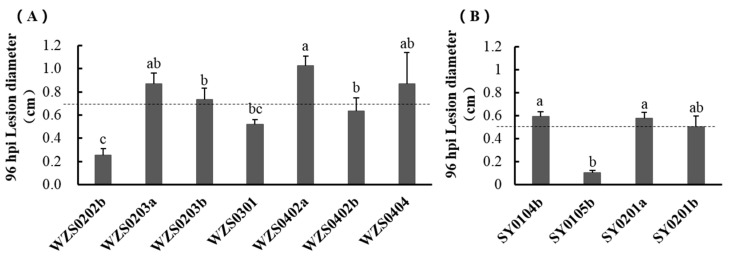
Pathogenicity of *C. fructicola* strains. (**A**) Pathogenicity of 7 *C. fructicola* strains from Wuzhishan. The dotted line is the mean lesion diameter. Error bars represent standard deviation. Letters above the bar indicate the significance of the difference in data pair comparison; the same letter indicates the difference is not significant. (**B**) Pathogenicity of 4 *C. fructicola* strains from Shaoyang. The dotted line is the median lesion diameter. Error bars represent standard deviation. Letters above the bar indicate the significance of the difference in data pair comparison; the same letter indicates the difference is not significant.

Results of the pathogenicity test of the Wuzhishan population are shown in Figure 2A. After 96 h from inoculation, the mean size of lesions caused by seven strains from Wuzhishan was 0.69 cm (dotted line in Figure 2A). Among the seven strains tested, WZS0202b was the less virulent, whereas WZS0402a and WZS0203a were the most virulent.

Results of the pathogenicity test of the Shaoyang population are shown in Figure 2B. After 96 h of inoculation mean size of lesions was 0.50 cm (dotted line in Figure 2B). strain SY0104b was the most virulent, while strain SY0105b was the least virulent.

Based on the results of the two strains of *C. fructicola*, WZS0402a and SY0104b from two distinct populations were selected for RNA sequencing.

### 2.3. Statistics of Differentially Expressed Genes

**Figure 3:**
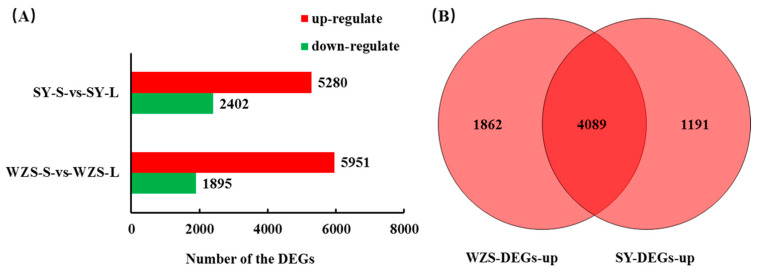
Overview of differentially expressed genes in *C. fructicola* strains. (**A**) Comparison of differential genes before and after infection. (**B**) Venn diagram of the number of up-regulated genes after infection.

Differentially expressed genes were screened using FDR (False Discovery Rate) ≤ 0.05 and |log_2_FC| ≥ 1 as thresholds, and then genes were manually eliminated if they had low expression levels (FPKM < 0.5), resulting in the selection of WZS-S-vs-WZS-L (Wuzhishan start vs. Wuzhishan later), SY-S-vs-SY-L. The number of up and down-regulated genes in the infection groups was tabulated using homologous spore samples as a control (Figure 3A). In the WZS-S-vs-WZS-L group, a total of 7846 differential genes were screened, 5951 of which expression was up-regulated (75.85%), and 1895 (24.15%) down-regulated. In the SY-S-vs-SY-L group screening, a total of 7682 differential genes were identified, 5280 (68.73%) of which were up-regulated, and 2402 (31.27%) were down-regulated. During the interaction stage of oil tea infected with *C. fructicola*, the number of up-regulated genes was significantly higher than that of down-regulated genes in the two representative strains. The number of up-regulated genes of *C. fructicola* from Wuzhishan after infection was higher than from the Shaoyang strains. Comparison of differentially expressed gene lists post-inoculation demonstrated that 4089 genes were up-regulated in both strains after 96 h of infection (Figure 3B). Overall, similarly expressed genes accounted for 68.7% (WZS) and 77.4% (SY) of up-regulated genes, respectively.

### 2.4. Real-Time PCR 

**Figure 4:**
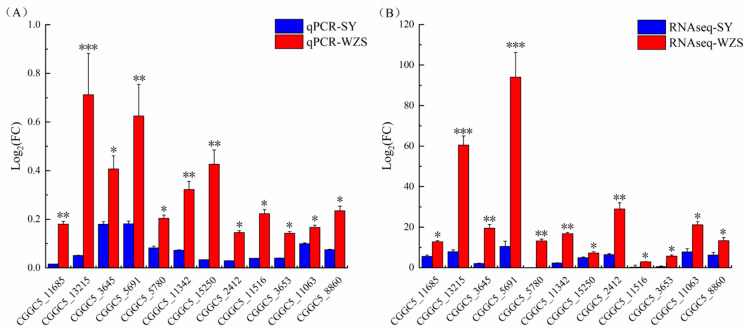
Expression verification of 12 up-regulated genes specific to the *C. fructicola* of WZS group. (**A**) qRT-PCR verification result of WZS-L-vs-SY-L. *** means difference between data of SY and WZS group is significant with *p*-value ≤ 0.01; ** means difference between data of SY and WZS group is significant with *p*-value ≤ 0.05; * means difference between data of SY and WZS group is significant with *p*-value ≤ 0.1. (**B**) RNAseq verification result of WZS-L-vs-SY-L. *** means difference between data of SY and WZS group is significant with *p*-value ≤ 0.01; ** means difference between data of SY and WZS group is significant with *p*-value ≤ 0.05; * means difference between data of SY and WZS group is significant with *p*-value ≤ 0.1.

In this case, 12 differentially expressed genes were selected for real-time PCR to verify the reliability of the Wuzhishan strain’s unique expression. The gene *Actin* was selected as the reference gene. Quantitative PCR results showed that most selected gene expression trends were consistent with transcriptome data (Figure 4). Furthermore, relative expression levels (log_2_FC value) were not significantly different from those of the RNA sequencing results, revealing that the impressive up-expression results of Wuzhishan strains are reliable.

### 2.5. Gene Ontology Enrichment Analysis

Gene ontology (GO) function enrichment analysis was performed on differentially expressed genes following infection of WZS-S-vs-WZS-L and SY-S-vs-SY-L groups. As shown in Table 1, GO terms were screened with *p*-value ≤ 0.01 as the threshold. Inoculation with the Wuzhishan representative strain enriched pre-ribosome, large ribosomal subunit, and organelle ribosome GO terms. Identified molecular functions were mainly related to structural, translation factor activity (RNA binding), and galactosidase activity. The biological processes were mainly associated with the glutamine family, amino acid metabolism pathway.

Screening of GO enrichment results of the Shaoyang group of strains demonstrated that enriched cell composition terms were related to ribosome precursors, large ribosome subunits, and organelle ribosomes. Molecular functions were mainly related to structural molecular activity and galactosidase activity. Identified biological processes were mainly related to amino acid metabolic processes and glycoprotein metabolic processes of the glutamine family. 

As shown in Table 1, the number of differentially expressed genes in the corresponding GO annotations in the Shaoyang group was significantly lesser than that in the Wuzhishan group. The two groups enriched molecular functions were related to structural molecular activity and galactosidase activity; however, the enriched RNA-bound translation factor activity of the Wuzhishan group was not significantly enriched in the Shaoyang group. Both groups have enriched GO biological processes related to glutamine-amide family amino acid metabolism. However, glycoprotein metabolism was enriched in the Shaoyang group and not in the Wuzhishan group. Of the two co-enriched annotations, genes associated with galactosidase activity and amino acid metabolism of the glutamine family were most similar.

### 2.6. KEGG Pathway Enrichment Analysis

Analysis of gene lists identified seven KEGG pathways (Q value ≤ 0.01) following inoculation with the Wuzhishan and Shaoyang populations (Table 2). Of identified GO pathways, ribosome (ko03010), arginine and proline metabolism (ko00330) were significantly enriched in both groups, indicating that arginine and proline metabolism play an important role in processes of infection of *C. fructicola*. KEGG pathways significantly enriched only in the Wuzhishan population included ribosome biogenesis in eukaryotes (ko03008), spliceosome (ko03040), RNA transport (ko03013), RNA polymerase (ko03020), and purine metabolism (ko00230). Pathways that were significantly enriched only in the Shaoyang group included phenylalanine metabolism and beta-alanine metabolism.

**Figure 5:**
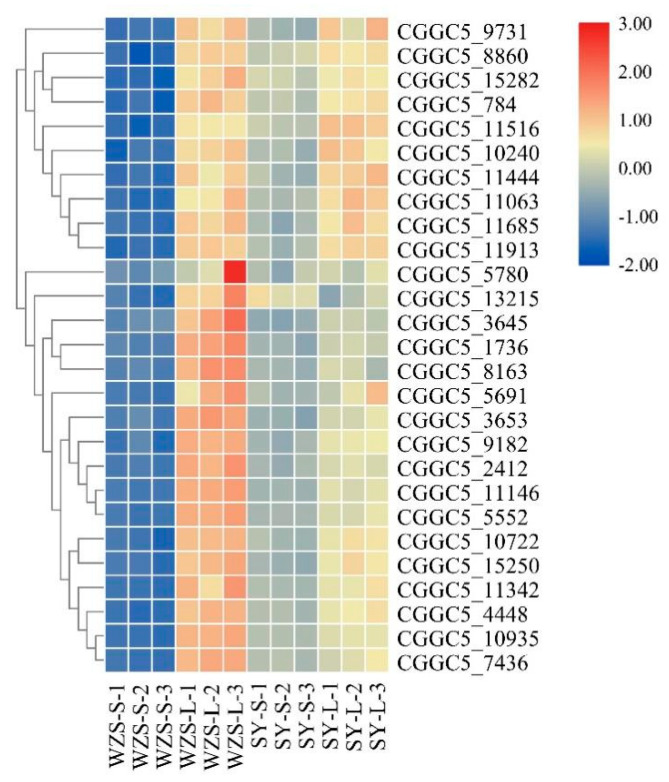
Heatmap of specific genes expression in the two *C. fructicola* strains from different sources. The heatmap was created using the TBtool (version 0.66837, [23]) function “heatmap” with default parameter setting and shows normalized FPKM values.

Comparison of differential genes associated with the purine metabolism pathway revealed that 35 differential genes were up-regulated in the Wuzhishan group and Shaoyang group. In comparison, the remaining 27 up-regulated genes in the Wuzhishan group were not up-regulated in the Shaoyang group. Figure 5 shown the heatmap of the 27 up-regulated genes specific to the WZS group. Of the up-regulated genes specific to the purine metabolism pathway following inoculation with the Wuzhishan population, 12 of the 27 genes are involved in regulating purine biosynthesis and catabolic metabolism (Table 3). This pathway’s related downstream products include guanylate synthase, adenylate deaminase, inosine cyclic hydrolase, and adenine phosphoribosyltransferase. The remaining 17 genes of the Wuzhishan population an up-regulated gene list is involved in RNA and DNA synthesis. 

## 3. Discussion

### 3.1. C. fructicola Collected in Wuzhishan Has a Stronger Ability to Destroy Oil Tea Leaf Tissue

The two regions selected for comparison of *C. fructicola* populations are approximately 7° in latitude apart. The average annual rainfall and temperature of Wuzhishan are higher than that of Shaoyang. However, Shaoyang experiences more significant temperature variation, with winter temperatures not suitable for the proliferation of *C. fructicola*. Based on the temperature data, we hypothesize that the anthracnose pathogen’s wintering time in Wuzhishan is transient and that infection initiation is earlier. 

A comparison of biological characteristics and pathogenicity of the two geographical populations of *C. fructicola* found that the number of lesions 96 hpi did not differ statistically. However, there was a significant difference in the diameter of the lesions between the two populations of *C. fructicola* 96 h after inoculation on detached leaves of oil tea. The spread of lesions on oil tea caused by the Wuzhishan population was more severe, indicating that *C. fructicola* from Wuzhishan had a more vital ability to kill oil tea leaf tissue. 

Based on the results of the phenotypic experiments, the two populations of *C. fructicola* had similar abilities to infect the host; however, their ability to further colonize the infected tissues differed.

### 3.2. Changes in C. fructicola Carbon Source Utilization on Oil Tea Leaf Tissues in the Late Infection Stage

RNA sequencing results demonstrated gene, GO term, and KEGG pathway enrichment related to the ribosomal activity for both populations. Enriched GO cell composition terms included ribosomes in both populations, which indicates that mitochondria and endoplasmic reticulum ribosomes might play essential roles in the infection progress and illustrates the active regulation of various proteins by pathogenic fungi after infection. 

GO functional enrichment analysis suggest that sugar conversion and utilization are exist and relatively active in the late stage of *C. fructicola* infection. After infection by fungi of hemi biotroph, carbohydrates in the apoplast are important carbon sources for pathogenic fungi. Proteins such as hexose transporters can absorb nutrients from host cell tissues or resist defensive immune stress in plants. Previous work has found that four hexose transporters (CgHxt1, CgHxt2, CgHxt3, and CgHxt5) of *Colletotrichum graminicola* can transport a variety of hexoses, including fructose, mannose, galactose, and xylose. The transporter genes *CgHxt2* and *CgHxt5* are only expressed in the vegetative phase based on dead plant tissue [24]. Another study showed that *MFS1* gene knockouts of *Colletotrichum lindemuthianum* had defects in the use of glucose, mannose, and fructose. Furthermore, semi-quantitative PCR analysis found that the *MFS1* gene was only up-regulated during the vegetative phase based on dead plant tissue 96 h after infecting the host [25]. Galactosidase is a class of enzymes that hydrolyze galactosyl bond-containing substances, and hydrolysis produces substances such as galactose, glucose, and fructose [26]. However, few studies exist on enzymes related to the molecular mechanisms of phytopathogenic fungi. It can be inferred that hydrolysis of galactosidase may be a new carbon source for *C. fructicola* after 96 h infection with oil tea.

### 3.3. Up-Regulation of Purine Metabolism May Help Improves Tissue Destroy Ability of C. fructicola

Combine the GO results with the expression, it was observed that ribosome-related genes of Wuzhishan and Shaoyang fungi strains were significantly up-regulated following infection. However, the number of differentially expressed genes associated with ribosome-related annotations in the Shaoyang strain was significantly less than that in the Wuzhishan strain. In addition, a comparison of GO analysis results of differentially expressed gene lists demonstrated enrichment in the activity of translation factors for the Wuzhishan strain but not the Shaoyang strain, indicating that the translation factor activity of the Wuzhishan strain was significantly higher during the process of ribosomal translation. The KEGG enrichment analysis indicated that the Wuzhishan strain was significantly enriched in signal pathways such as spliceosome, RNA transport, and RNA polymerase but not significantly enriched in the Shaoyang strain. Differences in transcriptional and translational activity between the two *C. fructicola* strains suggest that the Wuzhishan population is highly active and that enriched pathways and differentially expressed genes may be associated with its greater destructiveness.

**Figure 6:**
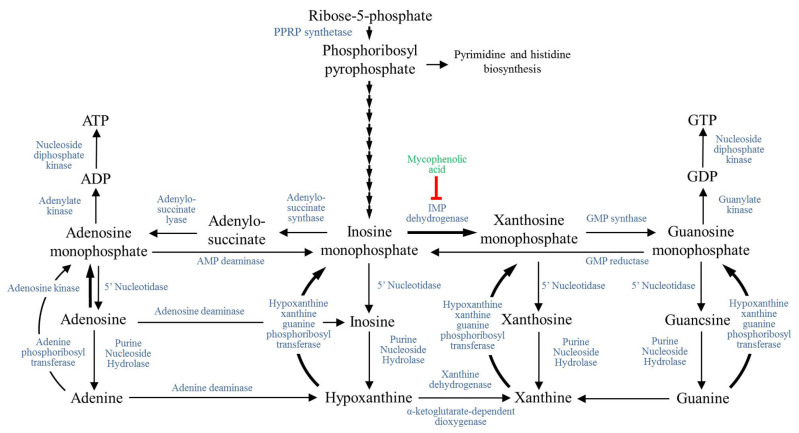
Components of the purine metabolic pathway [33]. The de novo synthesis pathway uses 5-phosphate ribose (Ribose-5P) as the raw material. It generates phosphoribosyl pyrophosphate (PRPP) via phosphoribosyl pyrophosphate kinase (PRS), which then synthesizes inosinic acid (IMP) through 10 consecutive reactions catalyzed by various enzymes. In the de novo synthesis pathway, IMP is converted into adenylate (AMP), or to xanthosine (XMP) and then guanylate (GMP), and finally to ATP and GTP. In the rescue pathway, free adenine and guanine in cells synthesize AMP and GMP under the action of adenine phosphoribosyltransferase (APT). The purine degradation pathway first converts purine nucleotides, such as AMP and GMP, into xanthine by various enzymes. Xanthine is finally degraded to produce uric acid.

Purine nucleotide metabolism is necessary for biological metabolic processes and the expression of genetic information and supports several physiological and biochemical reactions [27]. Purine in living organisms can be divided into adenine, guanine, xanthine, and hypoxanthine. Purines are essential nucleic acid and participate in protein translation, phosphate utilization, and energy metabolism [28]. Purine biosynthesis includes de novo synthesis and salvage pathway (Figure 6). Using KEGG enrichment analysis, we found that differentially expressed genes in the Wuzhishan strain were significantly enriched in processes related to purine metabolism, indicating that *C. fructicola* in the Wuzhishan group induces purine related genes associated with the nucleotide metabolic pathway after infection. We found that 33 differentially expressed genes in the Wuzhishan strain and Shaoyang strain were up-regulated following infection. In comparison, the remaining 27 of the 62 up-regulated genes were specific to the Wuzhishan strain. Meanwhile, there was no significant difference in the expression levels of these 27 genes in the Shaoyang strain before and after infection. In this case, 12 of the 27 genes specific to the Wuzhishan strain were involved in the regulation of purine biosynthesis and catabolic metabolism (Table 3), especially biosynthetic pathways. Of the 12 genes, four (*PRS5*, *ADE3*, *ADE17*, *ADE6*) are involved in regulating the biosynthesis of IMP in the purine de novo synthesis pathway, three (*ADSS*, *ADK*, *GUA1*) are involved in regulating ATP and GTP synthesis by IMP, one (*APT1*) participates in the regulation of purine rescue pathways, and four (*AAH1*, *ADA1*, *UAZ*, *NT5E*) participate in the regulation of purine degradation pathways.

Genes that were up-regulated explicitly in the Wuzhishan strain play essential roles in anabolic purine metabolism. Data show that the purine de novo synthesis pathway is directly related to pathogenic fungi’ growth, development, and pathogenicity [29]. Previously, it has been observed that the guanylate kinase and inosine-5′-phosphate lactate dehydrogenase (IMPDH) play vital roles in the GTP synthesis pathway. Knockdown of the Guanylate kinase gene, *MoGuk2*, in *Magnaporthe oryzae* led to reductions in the expansion of hyphae by the host [30]. The five active sites of the IMPDH coding gene, *MoIMD4*, regulate the pathogenicity of *M. oryzae*; thus, *MoIMD4* knockout mutants are less pathogenic than wild-type *M. oryzae* [31]. *ACD1* of *Fusarium graminearum* participates in the regulation of the conversion process of AMP to IMP. The knockout mutant of *ACD1* cannot form ascospores, and the expansion ability of the infection hyphae decreases in the host cells. Further analyses of phenotypes were performed in the knockout mutant of the *APT1*, *XPT1*, *AAH1*, and *GUD1* genes, revealed that the growth rate and pathogenicity of these four mutants were not significantly different from those of the wild type, which indicated when a de novo purine synthesis pathway exist in *F. graminearum*, the *APT1*, *XPT1*, *AAH1*, and *GUD1* genes involved in regulating the purine salvage pathway are not necessary for the growth and pathogenicity of *F. graminearum* [32]. Our analysis indicates that representative strains of Wuzhishan *C. fructicola* have more active purine metabolism, related to their greater pathogenicity.

## 4. Materials and Methods

*Colletotrichum fructicola* strains used for experiments were isolated from diseased oil tea leaves collected from Shaoyang, Hunan, and Wuzhishan. Hainan, which was stored in a freezing tube containing 30% glycerin aqueous solution at −80 °C. Four strains of *C. fructicola* were from Shaoyang, Hunan, and seven strains were from Wuzhishan, Hainan. The geographical and climatic characteristics of the two locations are shown in Table S1. 

### 4.1. Media Preparation

Potato dextrose agar medium (PDA medium) consisted of; 200 g peeled potatoes (from the local market in Changsha city, China) was added to 1000 mL water and heated for 20 min. The mixture was filtered through gauze, and 20 g glucose and 20 g agar were added to the filtrate. The mixture was autoclaved at 121 °C for 20 min. The potato dextrose broth medium (PDB medium) was produced without agar. One gram of yeast extract was added to 1 L of PDA or PDB medium to promote the production of conidia.

### 4.2. Inoculation Experiment on Leaves of Oil Tea

The pathogenicity of the tested strains was determined in vitro by the wound inoculation test using leaves of the oil tea, cv Huashuo. Young leaves were collected, petioles were sealed with paraffin wax, and then placed into a glass Petri dish with wet cotton pads added to maintain humidity. A sterilized needle was used to make four pinholes along each leaf surface, two on the left half and two on the right half of each leaf. There would be 12 leaves replicates and 64 pinholes in total. 

Strains from Wuzhishan (WZS) and Shaoyang (SY) were cultured on a PDA solid medium for 96 h. Hyphae pieces at the edge of several colonies were cut and transferred to 100 mL of prepared PDB medium and cultured on a shaker at 160 rpm and 28 °C for 48 h. The culture solution was filtered through three layers of microscope wipe paper into new centrifuge tubes and then centrifuged at 5000 rpm for 3 min. The supernatant was poured off, spores were rinsed twice with sterile water, and sterile water was added to reconstitute spores at a concentration of 1 × 10^9^ conidia mL^−1^. 

Leaves were inoculated with conidium suspension from WZS, and SY strains, the left half with WZS and right half of the leaf with SY and sterile water for control, each pinhole except controls was inoculated with 10 μL of conidium suspension at a concentration of 1 × 10^9^ conidia mL^−1^, for 3 replicate wounds per strain.

Petri dishes were sealed with parafilm and cultured in the dark at 28 °C for 96 h. Leaves were then removed, and the diameter of the diseased spots was measured and photographed.

### 4.3. Sampling of RNA

Prepare the conidium suspension of two representative strains from WZS and SY at a concentration of 1 × 10^9^ pcs/mL (The method is described in Section 4.2). Next, 1.5 mL of conidium suspension was placed into a 1.5 mL centrifuge tube and centrifuged at 12,000 rpm for 2 min. The supernatant was removed to obtain conidia samples immediately frozen with liquid nitrogen and stored in a −80 °C refrigerator.

Sterile water was added to the remaining spore fluid for dilution, and the number of spores was counted to adjust the concentration of spore fluid to 1 × 10^6^ pcs/mL. Petioles were sealed with wax and placed in a petri dish with wet absorbent cotton. The spore fluid was evenly sprayed onto the leaves, and then the petri dish was sealed. Leaves were incubated at 28 °C in the dark for 96 h. After incubation, leaves were cut and placed into collection tubes, then immediately frozen with liquid nitrogen and transferred to a −80 °C refrigerator. There were three biological replicates per sample.

### 4.4. RNA Sequencing and Data Analysis

Conidia samples of different strains and corresponding infected leaf samples constitute timeline controls for following RNA sequencing. The spores and lesion samples were sent to Genedenovo Biotechnology Co., Ltd., Guangzhou, China, for RNA extraction and RNA sequencing. The RNA extraction used OminiPlant RNA Kit (DNase I, CoWin Biosciences), and instruction can be found in supplementary material 2 and RNA sequencing was undergone with the HiSeq X Ten platform. Among the samples, the spore sample group number is identified as S (Start, the point of 0 hpi (hour post-inoculation), and the lesion sample group number is L (Later, the point of 96 hpi). There were three biological replicates per sample. Data analysis was performed online using the Omicsmart platform (Genedenovo Biotechnology Co., Ltd., Guangzhou, China; https://www.omicsmart.com/) (accessed on 27 December 2019). A genome of strain *Colletotrichum fructicola* Nara gc5 (GCA_000319635.2) was used for reference.

### 4.5. Real-Time PCR

The kits: FastQuant RT Kit with gDNase (TIANGEN Biotech Co., Ltd., Beijing, China); SuperReal PreMix Plus (SYBR Green) (TIANGEN Biotech Co., Ltd., Beijing, China) were used for the analysis of samples.

RNA Samples subjected to RNA-seq analysis used the FastQuant RT Kit with gDNase (TIANGEN) for first-strand cDNA synthesis. Five sequences for the RNA-seq consistency test were randomly selected from the differentially expressed gene data obtained. In this case, 12 unique up-expression consistency test sequences were from the gene data obtained by RNA sequencing of Wuzhishan strains. The NCBI primer-blast program was used to design all of the quantitative PCR primers. Table 4 provides some background information. Beijing Qingke Biotechnology Co., Ltd. (Beijing, China) performed the primer synthesis. The *Actin* gene, which was stably expressed in all RNA-seq samples and was highly expressed, was selected as the reference gene [34]. The real-time PCR test uses SuperReal PreMix Plus (SYBR Green). The reaction system consisted of 20 μL, including 1 μL of cDNA template (100 ng/L), and 0.75 μL for front and back primers. Data analysis was performed using QuantStudio ™ Design and Analysis Software (version 1.5.1, Thermo Fisher Scientific). For qRT-PCR data, relative expression log_2_FC was calculated using the Ct method and compared with RNA sequencing data. Each sample had three replicates.

### 4.6. Statistical Analysis

The diameters of *C. fructicola* strains from the Shaoyang population and Wuzhishan populations were measured after 96 h of incubation. Although pathogenicity in most pathogens was normally distributed, we used the median with interquartile range to describe the data distributions of the lesion diameters because of the small sample size. Nonparametric analysis was used to compare the medians of lesion diameters between two groups of strains. Differences between groups were examined with Mann–Whitney *U* test (α = 0.05). Rough dates were calculated using IBM SPSS Statistics 20 (IBM China Company Limited, Beijing, China), and Mann–Whitney *U* test was calculated with RStudio (Version 1.4.1106). 

## 5. Conclusions

In conclusion, we conducted a pathogenicity analysis of the *Colletotrichum fructicola* from two geographic areas, and for the first time, performed a transcriptome sequencing analysis of *C. fructicola* after inoculation. The results showed that the ability to colonize the plant tissues of the two geographic populations was divergent. Under the same background conditions, this difference may be related to the differentiation caused by the environmental pressure in areas where the two populations live. 

The transcriptome sequencing results found that the molecular responses of the two geographic populations of *C. fructicola* in the later stages of infection are significantly different, especially in the expression patterns of the genes related to nutrient scavenging and resistance to host defense mechanisms. The GO biological process of the representative strain from Shaoyang was significantly enriched in glycoprotein metabolism, but this process was not enriched in the representative strain from Wuzhishan. On the other hand, the Wuzhishan strain was significantly enriched in ribosome-related metabolic pathways and purine metabolic pathways, while the Shaoyang strain was not significantly enriched in these processes. Further research found that 12 up-regulated genes involved in the regulation of purine biosynthesis and catabolic metabolism in the Wuzhishan strain showed no difference in the Shaoyang strain. 

Combining this with the evidence that the Wuzhishan strain has greater pathogenicity than the Shaoyang strain, we concluded that the difference in purine metabolism might be the reason for the different pathogenicity of the two geographical populations. Furthermore, the Wuzhishan population is more active in transcriptional and translational activity, indicating that enriched pathways and differentially expressed genes might be related to its stronger destructiveness. This study clarified the similarities and differences of the two geographical populations’ metabolic processes and regulatory genes at the later stage of infection. It helped to understand the evolutionary and pathogenic mechanisms of *C. fructicola*.

## Figures and Tables

**Table 1:** Significantly enriched GO terms and related up-regulated genes in *C. fructicola* strains infected oil tea leaves.

GO ID	Term	All Genes with GO Annotation	DEGs with GO Annotation		*p* Value	
			WZS	SY	WZS	SY
GO:0030684	preribosome	88	84	58	6.17 × 10^−26^	1.42 × 10^−7^
GO:0015934	large ribosomal subunit	38	37	28	9.39 × 10^−13^	1.08 × 10^−5^
GO:0000313	organellar ribosome	48	44	36	2.61 × 10^−12^	2.71 × 10^−7^
GO:0005198	structural molecule activity	61	49	37	1.36 × 10^−10^	8.63 × 10^−5^
GO:0008135	translation factor activity, RNA binding	65	42	23	5.36 × 10^−5^	0.6050
GO:0015925	galactosidase activity	20	16	15	3.25 × 10^−4^	4.76 × 10^−4^
GO:0009064	glutamine family amino acid metabolic process	51	32	31	1.55 × 10^−3^	5.61 × 10^−4^
GO:0009100	glycoprotein metabolic process	30	11	18	0.7554	9.73 × 10^−3^

**Table 2:** Significantly enriched KEGG pathways and related up-regulated genes in *C. fructicola* strains infected oil tea leaves.

Pathway ID	Pathway	All Genes with Pathway Annotation	DEGs with Pathway Annotation		Q Value	
			WZS	SY	WZS	SY
ko03010	Ribosome	102	99	88	7.04 × 10^−30^	2.34 × 10^−20^
ko00330	Arginine and proline metabolism	79	51	50	3.45 × 10^−3^	1.07 × 10^−3^
ko00230	Purine metabolism	99	62	37	3.10 × 10^−3^	0.9999
ko03008	Ribosome biogenesis in eukaryotes	71	56	42	9.59 × 10^−8^	0.0231
ko03013	RNA transport	93	63	41	9.49 × 10^−5^	0.9079
ko03020	RNA polymerase	26	22	12	5.79 × 10^−4^	0.9727
ko03040	Spliceosome	88	68	40	1.33 × 10^−8^	0.7774

**Table 3:** 12 Up-regulated genes specific to the *C. fructicola* of WZS group related to purine synthesis and catabolism.

Gene ID	Gene	Description	Pathway Module
CGGC5_8860	APT1	Adenine phosphoribosyltransferase	-
CGGC5_5780	NT5E	5′-nucleotidase	-
CGGC5_5691	UAZ	Uricase	Purine degradation
CGGC5_3653	PRS5	Ribose-phosphate pyrophosphokinase	PRPP biosynthesis
CGGC5_3645	ADE6	Phosphoribosylformylglycinamidine synthase	IMP biosynthesis
CGGC5_2412	ADE17	IMP cyclohydrolase	IMP biosynthesis
CGGC5_15250	ADA1	AMP deaminase	-
CGGC5_13215	AAH1	Adenosine deaminase	-
CGGC5_11685	GUA1	GMP synthase	Guanine ribonucleotide biosynthesis
CGGC5_11516	SPCC830.11c	Adenylate kinase	Adenine ribonucleotide biosynthesis
CGGC5_11342	ADE3	Phosphoribosylformylglycinamidine synthase, partial	IMP biosynthesis
CGGC5_11063	NCU09789	Adenylosuccinate synthetase	Adenine ribonucleotide biosynthesis

**Table 4:** Primer-related information for real-time PCR.

Gene ID	Primer Sequence (5′→3′)	Tm
CGGC5_1052	F: GCTCAACCGCTTCCTGTCC R: GTTGAGGCTCTGCATGTTGG	60
MSTRG.12379	F: ATCCCAGCCAGTGGTCAAAG R: GACCTCAACACCGACTCCAG	60
CGGC5_14884	F: GAATCCCCAGGCACCTTTCA R: TTGAGCAGGATGCGAGAGC	60
CGGC5_8685	F: ACCTCAGGGCAACAACAACA R: AGGCTGTGGGAGTAGTAGGG	60
CGGC5_435	F: ACTTCCATCGTCTGGCAAGG R: ATAGGGCGCCGATGAAAGAG	60
CGGC5_11685	F: AATTGAGGAGGAGGCGAAGCAC R: AAGACTCGGATGGCCAAACCC	60
CGGC5_13215	F: TGCTCAGCCTGTGTCCCATCT R: AAACAACCGCCTCAATCTCCTT	60
CGGC5_3645	F: ACCAGGTTCAGATGGGAGAGTC R: AGCACCAGGGAAAGGAGAGAT	60
CGGC5_5691	F: ATGACCGTCTGCTGCCTGCT R: GTTGATGGGGTTCTCCTTCGC	60
CGGC5_5780	F: AGTTCAACAACGAGCCCATC R: CATAGCCAACAGAGCAAGCA	60
CGGC5_11342	F: ACCAGGTTCAGATGGGAGAGTC R: AGCACCAGGGAAAGGAGAGAT	60
CGGC5_15250	F: AATGCCCGGATGAGGTAGT R: TGTCGAATCGGTGAAAGGA	60
CGGC5_2412	F: AGAAGAAGAAGGGCGGAAAG R: TTGTAGGCGTAGCACACAGAGT	60
CGGC5_11516	F: CGTGAGGGTTGGGATGATGAA R: GAAGGACCACCACAAGGTCGA	60
CGGC5_3653	F: TACATCCAGCAGAACATACCCAA R: ACTCACCATCCAGGAAGCCA	60
CGGC5_11063	F: GCTACGACTTCCATCTTCTTCC R: AAACGTCCTCAGGTCCTCTCT	60
CGGC5_8860	F: GGGAAGGTAAAGCTCCAGAG R: GATGAAGAGGTAGCCAACGA	60
Actin	F: ATGTGCAAGGCCGGTTTCGC R: TACGAGTCCTTCTGGCCCAT	60

## Data Availability

The RNA sequencing data was submitted to NCBI SRA databases (https://www.ncbi.nlm.nih.gov/sra/?term=PRJNA644240) (accessed on 6 July 2020), and BioProject ID is PRJNA644240.

## Supplementary Materials

The following are available online at https://www.mdpi.com/article/10.3390/plants10122672/s1, Table S1: Geographical and climatic features of Wuzhishan and Shaoyang, Supplement material 2: OminiPlant RNA Kit (DNase I).
