# Supplementary material for: Transcriptome Analysis of Colletotrichum fructicola Infecting Camellia oleifera Indicates That Two Distinct Geographical Fungi Groups Have Different Destructive Proliferation Capacities Related to Purine Metabolism
Source: Plants (Basel). 2021 Dec 5;10(12):2672. doi: 10.3390/plants10122672 (PMC8708221; doi:10.3390/plants10122672)
Supplement: Supplementary file 1 [file plants-10-02672-s001.zip › plants-1401280 - supplementary materials 2.pdf]

Version No.: 01 / 2021

OminiPlant RNA Kit（DNase I）

Catalog No.: cw2598s (50 Preps)

Storage conditions: DNase I and 10×Reaction buffer is stored at - 20 ℃, and other components are stored at room temperature (15-30 ℃).

Product content

Component	CW2598S<br>50 preps
DNase I	1000 U
10×Reaction Buffer	1000 μL
Buffer RLS	40 mL
Buffer RW1	40 mL
Buffer RW2（concentrate）	11 mL
RNase-Free Water	10 mL
Spin Columns FS with Collection Tubes	50
Spin Columns RM with Collection Tubes	50
RNase-Free Centrifuge Tubes（1.5 mL）	50

RNA Yield

Plant sample (100mg)	Total RNA（μg）
Arabidopsis pod	~50
Soybean	~55
maize leaves	~55

Self-prepared reagent: β-Mercaptoethanol, absolute ethanol (special for newly opened or RNA extraction)

Preparation and important precautions before experiment

1. To prevent RNase pollution, the following suggestions should be paid attention to:
   1) Use RNase free plastic supplies and pipette tips.
   2) Operators should wear disposable masks and gloves, and change gloves frequently during the experiment.
2. Avoid repeated freezing and thawing of samples, otherwise it will affect the yield and quality of RNA extraction.
3. If precipitation appears in the buffer RLS, please dissolve it by heating and keep it at room temperature.
4. Please add β-Mercaptoethanol buffer RLS (1: 500) before use. The buffer RLS added β-Mercaptoethanol can be stored at room temperature for 1 month.
5. Before using Buffer RW2 for the first time, add absolute ethanol according to the instructions on the label of the reagent bottle.

Operation steps

1. Homogenization: Take 50-100mg of plant tissue, quickly grind it into powder in liquid nitrogen, and add 500μL Buffer RLS. (Please check if β-Mercaptoethanol is added before use), vortex and shake immediately to make it evenly mixed.
   Note: For materials with extremely rich water content, such as watermelon pulp, tomato, pear pulp, etc., more materials can be appropriately added, up to 200 mg; for starch-rich samples or mature leaves, buffer RLS can be appropriately added, up to a dosage of 700 μL.
2. Centrifuge for 2 minutes at 4°C, 12,000 rpm (~13,400×g).
3. Transfer the supernatant into the spin columns FS that have been loaded into the collection tube, centrifuge at 4 °C, 12,000 rpm for 1 minute, carefully suck the supernatant from the collection tube and transfer it to a new RNase free centrifuge tube (self-provided), and try to avoid the pipette tip from contacting the cell debris precipitation in the collection tube.
4. Slowly add absolute ethanol of 0.5 times the volume of supernatant, mix well (precipitation may occur at this time), and transfer the obtained solution and precipitate to the spin column RM that has been installed in the collection tube. Transfer the solution for two times if cannot finish the transformation at once. Centrifuge at 4°C, 12,000 rpm for 1 minute, discard the waste liquid, and put the spin column back into the collection tube.
5. Add 350 μL buffer RW1 to the spin column RM, centrifuge at 4 °C, 12,000 rpm for 1 minute, discard the waste liquid and put the spin column back into the collection tube.
6. Preparation of DNase I mixture: Take **52 μL RNase-Free Water,** add **8 μL 10×Reaction Buffer** and **20 μL DNase I (1 U/μL),** mix well, and prepare a reaction solution with a final volume of 80 μL.
7. Add **80 μL DNase I mixture** directly to the spin column and incubate at 20-30 ℃ for 15 minutes.
8. Add 350 μL buffer RW 1 to the spin column RM, centrifuge at 4 °C, 12,000 rpm for 1 minute, discard the waste liquid and put the spin column back into the collection tube.
9. Add 500 μL buffer RW2 to the spin column RM (check whether absolute ethanol is added before use), centrifuge at 4 ℃, 12,000 rpm for 1 minute, discard the waste liquid and put the spin column back into the collection tube.
10. Repeat step 9.
11. Centrifuge at 4 °C, 12,000 rpm for 2 minutes.
    Note: the purpose of this step is to remove the residual ethanol in the spin column, which will affect the subsequent enzymatic reactions (enzyme digestion, PCR, etc.).
12. Put the spin column RM into a new RNase free centrifuge tubes (1.5 mL) and drop 30-50μL RNase free water to the middle of the adsorption membrane, let stand at room temperature for 2 minutes, centrifuge at 4 °C 12,000 rpm for 1 minute. The obtained RNA solution should be stored at - 70 ℃ to prevent degradation.

Note:

1) the volume of RNase free water shall not be less than 30μL. Too small volume affects the recovery rate.
2) If you want to increase RNA production, you can use 30-50μL RNase free water and repeat step 12.
3) If the RNA concentration is to be increased, the obtained solution can be added to the spin column again and repeat step 12.
