# Supplementary material for: Transcriptome Analysis of Colletotrichum fructicola Infecting Camellia oleifera Indicates That Two Distinct Geographical Fungi Groups Have Different Destructive Proliferation Capacities Related to Purine Metabolism
Source: Plants (Basel). 2021 Dec 5;10(12):2672. doi: 10.3390/plants10122672 (PMC8708221; doi:10.3390/plants10122672)
Supplement: Supplementary file 1 [file plants-10-02672-s001.zip › plants-1401280 - Table S1.pdf]

**Table S1.** Geographical and climatic features of Wuzhishan and Shaoyang.

| Items | Hainan Wuzhishan | Hunan Shaoyang |
| --- | --- | --- |
| latitude and longitude | East longitude 109°19′~109°44′ North latitude 18°38′~19°02′ | East longitude 109°49'~112°57' North latitude 25°58'~27°40' |
| Climate type | Tropical ocean monsoon climate | Subtropical monsoon humid climate |
| Average annual temperature | 22.4 °C | 15 °C ~22 °C |
| The highest temperature in history (1959–2019) | 35.9 °C | 38 °C |
| The lowest temperature in history (1959–2019) | 11 °C | −22 °C |
| Average annual rainfall | 2444 mm | 1368 mm |
| Climate diversity | The large temperature difference between day and night; high temperatures throughout the year; Heavy rainfall in summer and extremely short spring and autumn. | The mild climate, distinct four seasons, significant seasonal temperature difference; sufficient rainfall, heavy rain in spring and summer; often dry in summer and autumn |
